# Ischemic Colitis after Colonoscopy with Bisacodyl Bowel Preparation: A Report of Two Cases

**DOI:** 10.1155/2020/8886817

**Published:** 2020-11-26

**Authors:** Chris Shamatutu, Daljeet Chahal, Isabella T. Tai, Peter Kwan

**Affiliations:** ^1^Department of Medicine, University of British Columbia, Vancouver, Canada; ^2^Department of Medicine, Division of Gastroenterology, University of British Columbia, Vancouver, Canada

## Abstract

**Background:**

Colonoscopy is widely used for the diagnosis and management of colorectal disease and requires adequate bowel preparation. Ischemic colitis is a form of intestinal ischemia that presents with abdominal pain, diarrhea, and hematochezia. Risk factors include advanced age, cardiovascular disease, and diabetes. Both colonoscopy and bisacodyl bowel preparation have been described as rare causes of ischemic colitis with less than 35 cases collectively in the literature. Our review found that of these cases, there exists significant heterogeneity within individual patient characteristics. The majority of the cases are managed conservatively without complications or sequela. Due to the risk of ischemic colitis, the FDA has withdrawn bisacodyl bowel preparations from use in the USA. Bisacodyl bowel preparations are still used in Canada.

**Cases:**

Here, we present two cases of ischemic colitis in previously healthy women aged 57 and 69 who underwent screening colonoscopy using bisacodyl bowel preparation. Both were treated conservatively without complications.

**Conclusion:**

Thus far, there has been one documented case of ischemic colitis following colonoscopy with bisacodyl bowel preparation; here, we present two additional cases with one case occurring without the presence of known risk factors for ischemic colitis. Our literature review finds that there is limited evidence surrounding bisacodyl as a causative agent of ischemic colitis. Cases often contain confounding variables such as the presence of known risk factors for ischemic colitis. Our report aims to highlight the need for a more comprehensive analysis evaluating the safety of bowel preparations as well as increasing the clinical awareness surrounding the rare risk of colonoscopy-induced ischemic colitis.

## 1. Introduction

Colonoscopy is a common modality for investigation and management of diseases of the colorectal tract and remains the gold standard for diagnosis and prevention of colorectal cancer [1]. Bowel preparation is required prior to colonoscopy to facilitate adequate endoscopic visualization of the mucosa. High-volume (4 L) polyethylene glycol (PEG) is a well-established, safe, and effective colon cleanser; however, up to 38% of patients do not complete this bowel preparation due to the large volume of ingested solution required and poor taste [2]. Inadequate bowel preparation occurs in up to 25% of colonoscopies [3] and results in a lower adenoma detection rate, longer colonoscopy time, and higher cost due to the need for repeat colonoscopy [4, 5].

Various preparations have been formulated in attempts to decrease the volume of solution ingested including the addition of bisacodyl, a colonic motility laxative. A combination of low-volume (2 L) PEG with 20 mg bisacodyl was first approved for use in the United States as bowel preparation in 2004. Case reports documenting episodes of ischemic colitis (IC) following the use of bisacodyl 20 mg/PEG 3350 bowel preparations resulted in the Food and Drug Administration (FDA) issuing a black box warning on these preparations in 2006. The bisacodyl 20 mg/PEG 3350 and bisacodyl 10 mg/PEG 3350 bowel preparations were eventually withdrawn from the United States market by the FDA in 2007 and 2010, respectively, due to “Reasons of Safety or Effectiveness.” In Canada, the formulation Bi-Peglyte (bisacodyl 15 mg/PEG 3350) is still in use and has not been withdrawn despite these findings.

Advanced age is the strongest risk factor for IC with 90% of individuals greater than age 60 [6]. Additional identified risk factors include hypertension, cardiovascular disease, and diabetes mellitus [7, 8]. Interestingly, colonoscopy itself has been described in the literature as a cause of IC with 25 studies identified globally [9]. In these case reports, we describe two cases of IC following colonoscopy with Bi-Peglyte (15 mg Bisacodyl/PEG 3350) used as bowel preparation. We subsequently conducted a review of the literature surrounding bowel preparation and colonoscopy-induced IC.

### 1.1. Case Presentation 1

A 57-year-old healthy female underwent colonoscopy in the context of a positive fecal immunohistochemistry test (FIT) of 77 ng/mL. Her medical history was only significant for a remote caesarean section. Her family history was significant for her father having colonic polyps at the age of 70. The patient also endorsed occasional NSAID use. The patient had used Bi-Peglyte solution for bowel preparation.

The patient was sedated before and during the procedure with a total of 3 mg of midazolam and 100 mcg of fentanyl. CO_2_ was used to insufflate the colon. The colon was quite tortuous and required repositioning of the patient on to her back and right side. Application of counter pressure was also used to reach the cecum. Bowel preparation was suboptimal, and fecal material clogged the endoscope numerous times. Extensive lavage and suction were required during withdrawal. A 1.2 cm pedunculated polyp in the descending colon was injected with methylene blue and completely removed as 2 pieces by hot snare polypectomy. Withdrawal time was 15 minutes. Overall, the patient tolerated the procedure well. Pathology of the polyp demonstrated a tubulovillous adenoma with low-grade dysplasia.

**Figure 1:**
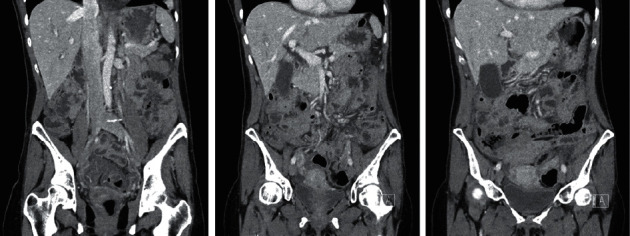
Computed tomography (CT) images demonstrated diffuse large bowel thickening consistent with ischemic colitis.

**Figure 2:**
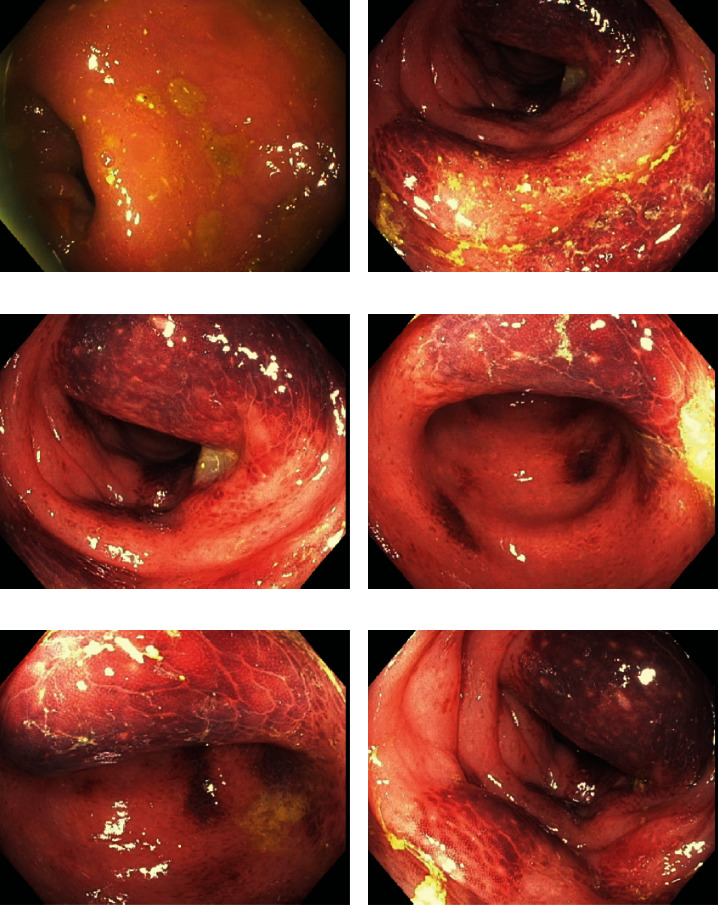
Endoscopic images up to midsigmoid demonstrating patchy, dusky areas of mucosa with submucosal swelling and erythema but no frank ulcerations.

The patient subsequently presented to the emergency department (ED) 2 days after colonoscopy with severe abdominal pain but no bleeding or diarrhea. Initial bloodwork was unremarkable other than an elevated lactate of 5. A CT abdomen with IV contrast demonstrated diffuse large bowel thickening with severe involvement of the sigmoid and small volume of pelvic ascites (Figure 1). There was no hemoperitoneum, pneumoperitoneum, or bowel obstruction. The patient underwent an urgent flexible sigmoidoscopy. Starting at the rectosigmoid junction, there were patchy, dusky areas of mucosa with submucosal swelling and erythema but no frank ulcerations (Figure 2). The endoscope was only advanced to the midsigmoid, and biopsies were not taken due to the fear of inducing more complications. The proximal extent of the changes was not determined.

A diagnosis of IC was made. She ultimately deteriorated hemodynamically and required placement of central venous access for vasopressors. She was then admitted to the high acuity unit (HAU) and monitored in anticipation of possible colectomy. Interestingly, her blood cultures were positive for group A *Streptococcus* (GAS). This bacteremia was thought acquired due to an occupational hazard and likely unrelated to her IC. She stated that she had experienced symptoms of pharyngitis several weeks ago. A CT of the neck ruled out an abscess and an echocardiogram ruled out infective endocarditis. Over the next few days, the patient's abdominal pain subsided, and she recovered hemodynamically without the need for surgery. She was discharged 6 days later with oral antibiotics for her bacteremia.

### 1.2. Case Presentation 2

**Figure 3:**
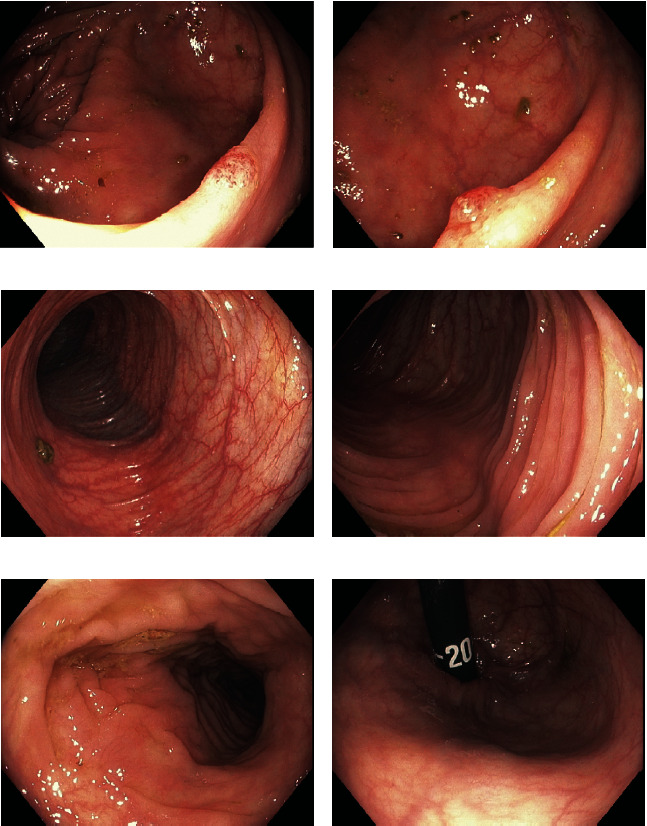
Initial endoscopic images from surveillance colonoscopy 6 days prior to the development of ischemic colitis symptoms. Images are in similar locations to their corresponding letters in Figure 4 for comparison.

A 69-year-old healthy female underwent a surveillance colonoscopy Figure 3. Her medical history was only significant for caesarean section, hysterectomy, oophorectomy, and irritable bowel syndrome. Her family history was significant for colorectal malignancy. She endorsed daily NSAID use in the form of ibuprofen and ASA for back pain. The patient had used Bi-Peglyte solution for bowel preparation.

The patient was sedated before and during the procedure with a total of 3 mg of midazolam and 75 mcg of fentanyl. Carbon dioxide (CO_2_) was used to insufflate the colon. The colonoscope was advanced without difficulty to the cecum. Bowel preparation was good. Two flat polyps of 2 mm size were removed by cold snare polypectomy in the ascending colon. Two flat, larger polyps of 1 cm size were removed from the hepatic flexure and transverse colon using methylene blue and cold snare polypectomy. Moderate diverticulosis was noted in the sigmoid colon. Pathology demonstrated normal mucosa, one tubular adenoma, and two sessile serrated lesions without dysplasia.

**Figure 4:**
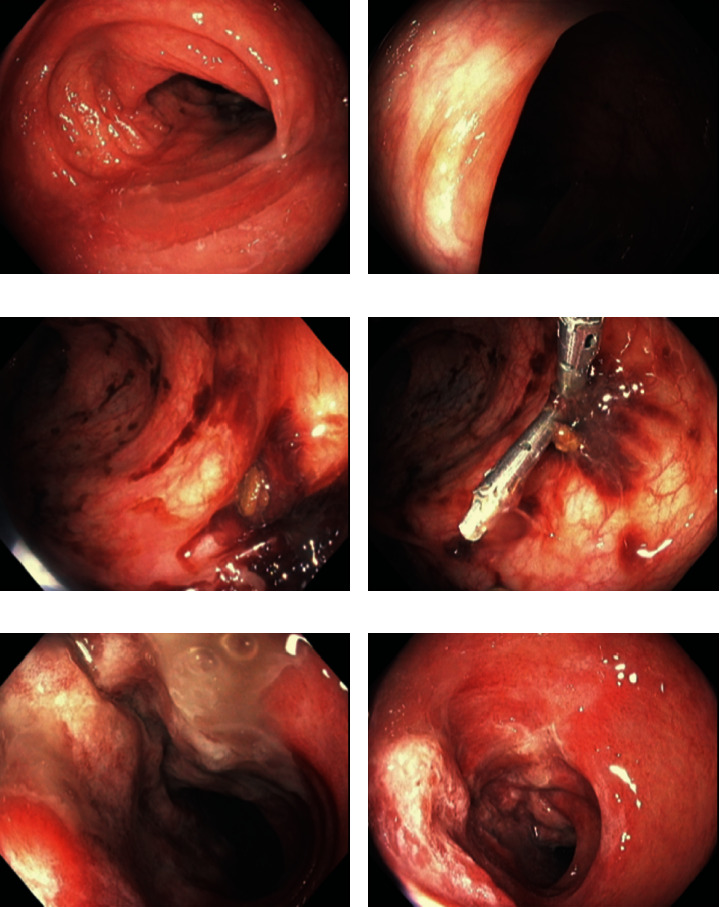
Endoscopic images demonstrating the noncircumferential segment of erythema and friability without submucosal edema or ulceration (1–6). Site of increased blood where a clip was placed (4).

The patient developed abdominal pain and small volume rectal bleeding 6 days after the colonoscopy. There was no peritonitis. She underwent an urgent flexible sigmoidoscopy the next day. Starting at the distal sigmoid, a 9 cm, noncircumferential segment of erythema and friability consistent with ischemia was noted (Figure 4). There was no submucosal edema or ulceration. There was a small area of increased blood, although no active bleeding, onto which a single clip was placed. No biopsies were taken. A CT scan completed 7 days after the flexible sigmoidoscopy did not demonstrate any features consistent with ischemia. The patient did well clinically and did not require hospital admission.

## 2. Discussion

We have presented two cases of IC following colonoscopy with Bi-Peglyte bowel preparation in patients with no known risk factors. Our first patient's course deteriorated with hemodynamic instability requiring vasopressors, and she was concomitantly found to have GAS bacteremia and treated accordingly. Her clinical condition improved with conservative management, and she experienced resolution of symptoms after 6 days. She was discharged in stable condition on oral antibiotics. Our second patient did well with supportive management and did not require hospital admission.

IC manifests most commonly as hematochezia, colicky abdominal pain, and diarrhea as a result of transient hypoperfusion to the colon [10]. If suspected, IC requires urgent endoscopy to confirm the diagnosis and evaluate the extent of bowel wall injury ideally within 48 hours. IC is predominantly self-limiting and managed with conservative measures including intravenous fluids, analgesia, and bowel rest in 83–92% of cases [11, 12]. Necrosis and perforation of the colonic wall is a known complication of severe IC requiring emergent surgical intervention. The need for intervention is an indicator of poor prognosis with an estimated operative mortality of 40% [13].

Well-defined factors for IC include age, hypertension, coronary artery disease, dyslipidemia, diabetes mellitus, peripheral vascular disease, chronic obstructive pulmonary disease, nephropathies, and atrial fibrillation [8]. In younger individuals without traditional risk factors, IC has been associated with autoimmune diseases, coagulopathies, endurance exercise, illicit drugs, and medications such as oral contraceptives, as well as various bowel preparation agents and colonoscopy itself as we describe. Our first case of IC was found to have concomitant GAS bacteremia; this has not been documented as a risk factor in the literature prior, and we proposed that it was unrelated to the episode of IC given the timing of IC postcolonoscopy. However, it should be noted that an association with *E. coli* O157 : H7 and IC has been described [14]. History of C-section has not been documented as a risk factor.

IC secondary to laxative use was first described in 1997 by Oh et al. who documented two cases of IC following hyperosmotic laxative ingestion as bowel preparation prior to colonoscopy [15]. The mechanism of action is thought to be multifactorial and related to the laxative effect of decreased intravascular volume as well as colonic hypermobility leading to increased intraluminal pressures and subsequent hypoperfusion [16, 17]. A review of the literature was conducted identifying similar cases of IC with laxative use prior to or in the absence of colonoscopy; a summary of these results is presented in Table 1. There are 12 documented cases of IC following laxative use in individuals ≤70 years of age. Common risk factors for IC were present in 6 cases. Laxative use indications in these reports included bowel preparation prior to colonoscopy (*N* = 8), bowel preparation prior to non-GI surgery (*N* = 1), and constipation (*n* = 3). Bisacodyl was the most commonly identified laxative and was implicated in 58% of cases (*n* = 7) with doses ranging from 5 mg to 20 mg and a time of onset ranging from 2 to 24 hours. Of the 7 cases of bisacodyl-associated IC, 2 were after bisacodyl tablet use for constipation and 5 were after bisacodyl/PEG 3350 for bowel preparation. Of these 5 cases, 2 are brief abstracts with limited clinical information and 3 had known risk factors for IC (hypertension, older age, and contraceptive use as well as constipation). Symptoms of abdominal pain and hematochezia were present in 66% of cases. Amongst all cases, management was conservative without complications (*n* = 12), and in most cases, patients were discharged home within 48–72 hours (*n* = 11).

Currently, the bowel preparation Bi-Peglyte and bisacodyl are still in use in Canada. Standard bowel preparation for inpatient colonoscopy at our institution involves a Bi-Peglyte equivalent, consisting of 15 mg of bisacodyl followed by 2 L of PEG 3350 in the evening prior to colonoscopy. This is followed by an additional 2 L of PEG 3350 in the morning of endoscopy as needed. Bisacodyl enemas also remain a part of our institutions constipation bowel protocol. The FDAs withdrawal of Halflytely bowel preparation with 10 mg and 20 mg of bisacodyl over concerns of IC is based on a limited number of case reports. Given the growing number of recognized factors that contribute an increased risk of IC [8] causation between bisacodyl and IC is debatable. The 2010 FDA clinical review gave an estimated IC incidence rate of 1/100,000 for the 20 mg preparation [25]. The risk of IC for the 10 mg preparation is described as “markedly lower” without a numerical estimate and is based on 3 case reports. However, two of these episodes were external reports where patients consumed an unknown dosage of additional bisacodyl in addition to the 10 mg contained in the Halflytely preparation. Only one of the three cases was identified in a clinical trial, a 55-year-old woman who was found to have IC one month postcolonoscopy. Interestingly, similar to our report, she required ICU admission secondary to unstable urosepsis which was presumably bacterial in origin. *E. coli* is the most common pathogen responsible for urinary tract infections [26] and as aforementioned has been associated with IC highlighting the difficulty in assessing the validity of the causal relationships presented. Based on our review, bisacodyl at current doses in bowel preparation is unlikely to be strongly associated with IC.

IC as a rare postcolonoscopy or sigmoidoscopy complication, irrespective of bowel preparation used, was first described in 1990 by Wheeldon and Grundman who documented a case of IC in an individual with systemic lupus erythematosus 24 hours after surveillance colonoscopy [27]. Proposed mechanisms of colonoscopy-induced IC include elevated intraluminal pressures and barotrauma to the colonic wall impairing arterial oxygen delivery [28–30]. A review of the literature was conducted for cases of IC following colonoscopy in individuals ≤70 years of age, and a summary of these results is presented in Table 2. There are 24 documented cases of IC following colonic endoscopy use in individuals ≤70 years of age; 19 of these are found in English literature. Of these 19, common risk factors for IC were present in 5 cases and included autoimmune disease (*n* = 3), advanced age with hypertension (*n* = 2), and chronic obstructive pulmonary disease (*n* = 1). Screening and surveillance were the indications for colonoscopy in 53% of cases. Bowel preparation regimens prior to colonoscopy were poorly described and varied across studies. Bisacodyl was used as bowel preparation in 2 cases. A description of colonoscopy characteristics including difficulties encountered was similarly lacking across studies. The time of onset postcolonoscopy varied from 1 hour to 6 days. Management of IC was conservative in 84% of cases. There was significant heterogeneity in the time from initial presentation of IC to resolution of symptoms and subsequent discharge (2–22 days).

Colonoscopy is thought to play a role in the development of IC through its effect of elevating intraluminal pressures and barotrauma to the colonic wall, thus reducing colonic blood blow and impairing oxygen delivery [28–30, 38, 42]. Decreasing procedural time reduces the amount of time the colon experiences luminal distention and has been proposed to reduce the risk of IC. This has not been well studied, and in fact, our review found that the total procedure time is rarely reported (Table 2). In both of the cases, we presented used CO_2_ as an insufflation agent. Due to its rapid absorption and vasodilatory properties, the utilization of CO_2_ over room air as an insufflation agent has been proposed to decrease the risk of IC through decreased duration of distension and improved perfusion, respectively [37, 43]. While multiple randomized controlled trials and meta-analysis comparing room air to CO_2_ insufflation have shown CO_2_ insufflation to decrease postprocedural abdominal distension and pain, its role in decreasing episodes of ischemic colitis relative to room air insufflation has not been well studied [44, 45].In conclusion, IC after bisacodyl bowel preparation or colonoscopy is a rare complication. Currently used doses of bisacodyl in bowel preparations are unlikely to be independently associated with IC, but may contribute in at risk patients. Our first case is one of 3 cases with a complicated course. Novel to this case is the presence of GAS bacteremia with a history of pharyngitis, the significance of which is unknown to date. Case reports of this rare event thus far contain limited data on bowel preparation specifics and technical details of the colonoscopy itself. Improved documentation of such variables in future reports is needed to further characterize this event.

## Figures and Tables

**Table 1:** Literature review results of ischemic colitis induced by bowel cathartics. Search run, August 2019.

First author, year	Age	Sex	Risk factors	Laxative Type Dose Reason	Onset to symptoms (hours)	Symptoms	Diagnosis	Complications	Treatment	Resolution
Oh [15], 1997	69	F	Age, HTN, and DM	Magnesium citrate NA Bowel preparation	1 hour after magnesium citrate	Abdopain and hematochezia	Colonoscopy	None	Conservative	D/C 7 days
Oh [15], 1997	66	F	Age and CAD	Sodium phosphate NA Bowel preparation	0.5 hours after sodium phosphate	Abdopain, nausea, emesis, and diarrhea	Colonoscopy	None	Conservative	D/C 2 days
Lopez [18], 2005	19	M	Constipation	Bisacodyl 5 mg Constipation	Several hours after bisacodyl	Abdopain, hematochezia, and fever	Colonoscopy and CT	None	Conservative	D/C 2 days
Lopez [18], 2005	33	F	Constipation Sertraline	Bisacodyl 10 mg Constipation	6 hours after bisacodyl	Abdopain and hematochezia	Colonoscopy	None	Conservative	D/C 2 days
Vaizman [19], 2007	56	M	HTN	Bisacodyl/PEG 3350 20 mg/NA Bowel preparation	After bisacodyl/PEG (unspecified)	Abdopain, hematochezia, and diarrhea	Colonoscopy	NA	NA	NA
Lee [20], 2008 (no English text)	39	F	None	Magnesium citrate 250 ml Bowel preparation	24 hours after magnesium citrate	Abdopain and hematochezia	Colonoscopy	None	Conservative	D/C 3 days
You [21], 2009 (no English text)	70	F	Age, COPD, and chronic constipation	SF-PEG 4 L Bowel preparation	0.5 hours after SF-PEG	Abdopain	Colonoscopy	NA	NA	NA
Baudet [16], 2010	68, 70*∗*	M	Age	Bisacodyl/PEG 10 mg/2 L Bowel preparation	2 hours after bisacodyl	Abdopain and hematochezia	Colonoscopy	None	NA	NA
Dholakia [22], 2011 (abstract only)	37	F	None	Bisacodyl/PEG 3350 NA Bowel preparation	24 hours after bisacodyl/PEG 3350	NA	Colonoscopy	None	NA	NA
Ajani [17], 2012	54	F	Chronic constipation OCP (estradiol 0.5 mg daily)	Bisacodyl 15 mg Bowel preparation for non-GI surgery	6 hours after bisacodyl	Abdopain, hematochezia, and diarrhea	Colonoscopy and CT	None	Conservative	NA
Sherid [23], 2013	54	F	DM, HTN, dyslipidemia, and chronic constipation	Lubiprostone 24 mcg Constipation	2 hours after lubiprostone	Abdopain, nausea, clear emesis, and hematochezia	Colonoscopy	None	Stopping lubiprostone	D/C 4 days
Behzadi [24], 2015 (abstract only)	64	F	None	Bisacodyl/PEG 3350 15 mg/2 L Bowel preparation	Several hours after bisacodyl/PEG 3350	Abdopain, nausea, clear emesis, and diarrhea	Colonoscopy and biopsy	None	Conservative	D/C several days

*∗*Recurrence in same individual 2 years later with same bowel preparation and same clinical course. Abdopain, abdominal pain; HTN, hypertension; DM, diabetes mellitus; CAD, coronary artery disease; COPD, chronic obstructive pulmonary disease; OCP, oral contraceptive pill; NA, not available; D/C, day of discharge; CT, computerized tomography. Conservative treatment in all cases consisted of IV fluids ± antibiotics.

**Table 2:** Literature review results of ischemic colitis following colonoscopy. Search run August 2019.

First author, year	Age	Sex	Risk factors	Bowel Preparation Type Dose	Initial endoscopy characteristics Indication for endoscopy Type of endoscopy Difficult endoscopy (Y/N) Time	Onset to symptoms (hours)	Symptoms	Diagnosis	Complications	Treatment	Resolution
Wheeldon [27], 1990	59	F	SLE	NA NA	Surveillance Colonoscopy No NA	24 hours	Abdopain, vomiting, diarrhea, and hematochezia	Colonoscopy	None	Conservative	D/C 6 days
Church [31], 1995	45	F	MCTD Methotrexate and prednisone	NA	Hematochezia Flexible sigmoidoscopy Yes: “Difficult, severe pain” NA	24 hours	Abdopain and fever	Colonoscopy	Peritonitis	Loop sigmoid colostomy	NA
Cremers [32], 1998	44	F	None	PEG 3350 4 L	Abdominal pain Colonoscopy No NA	48 hours	Abdopain and hematochezia	Colonoscopy	None	Conservative	D/C 2 days
Versaci [29], 2005	43	F	SLE	NA	Altered bowel habits Colonoscopy, polypectomy No NA	4 hours	Abdopain, hematochezia, mucous, and diarrhea	Colonoscopy	None	Conservative	D/C 12 days
Vaizman [19], 2007	78	F	HTN, age	Bisacodyl/PEG 3350 NA	Surveillance Colonoscopy NA NA	Later that day	Abdopain, hematochezia, and diarrhea	Laparotomy	Necrotic sigmoid with perforation	Left hemicolectomy	NA
Arhan [28], 2009	25	F	None	Calcium sennoside sodium phosphate enema	Constipation Colonoscopy 25 mins NA NA	1 hour	Abdopain and hematochezia	Colonoscopy	None	Conservative	D/C 5 days
Kao [30], 2009	55	M	None	NA	Screening Colonoscopy NA NA	NA	Abdopain and hematochezia	Colonoscopy	None	Conservative	NA
Dong [33], 2009	54	F	None	NA	Abdominal pain Colonoscopy NA NA	3 hours	Abdopain and hematochezia	Sigmoidoscopy and CT	None	Conservative	NA
Singh-Ranger [34], 2011	49	F	None	Sodium picosulphate 20 mg	Abdominal pain and loose stool Colonoscopy NA NA	Later that evening	Abdopain and fever	CT	None	Conservative	NA
Cheng [35], 2012	NA	M	None	Sodium phosphate 90 mL	Screening Colonoscopy NA NA	Several hours	Abdopain, hematochezia, and diarrhea	Colonoscopy and CT	None	Conservative	D/C 7 days
Sapmaz [36], 2014	26	F	None	NA	Chronic diarrhea Colonoscopy NA NA	10 hours	Abdopain and hematochezia	Colonoscopy and CT	None	Conservative	D/C 5 days
Lee [37], 2014	47	F	None	PEG 3350 4 L	Screening Colonoscopy and polypectomy NA NA	7 hours	Abdopain and hematochezia	Colonoscopy and CT	None	Conservative	D/C 9 days
Lee [37], 2014	40	M	None	PEG 3350 4 L	Screening Colonoscopy No NA	18 hours	Abdopain and hematochezia	Colonoscopy and CT	None	Conservative	D/C 9 days
Silva [38], 2014 (abstract only)	67	F	COPD	NA	Screening Colonoscopy NA NA	6 hours	Abdopain and hematochezia	CT	None	Conservative	D/C 5 days
Hai-Bo Zhou [39], 2015	30	F	None	NA	Abdopain Colonoscopy NA NA	48 hours	Abdopain and hematochezia	Colonoscopy	None	Conservative	D/C 7 days
Omar [40], 2015	70	F	Age, HTN, and dyslipidemia	PEG 3350 NA	Screening Colonoscopy No 30 mins	The next day	Abdopain and hematochezia	Colonoscopy and CT	None	Conservative	NA
Solanke [41], 2016	43	M	None	PEG 3350 4 L split	Constipation Colonoscopy NA NA	6 hours	Abdopain and hematochezia	Colonoscopy and CT	None	Conservative	D/C 2 days
Zizzo [9], 2016	43	F	None	PEG 3350 4 L	Screening Colonoscopy Yes: forced exploration, with insufflation 37 mins	12 hours	Abdopain, fever, and hematochezia	CT, laparoscopy, and laparotomy	Peritonitis Transmural infarct with necrosis	Left hemicolectomy and terminal colostomy	D/C 22 days
Current case 1, 019	57	F	None	Bisacodyl/PEG 3350 15 mg/2 L	(+) positive FITT Colonoscopy Yes: tortuous NA	48 hours	Abdopain	Flexible sigmoidoscopy and CT	Hemodynamic instability GAS bacteremia	Conservative	D/C 6 days
Current case 2, 2019	69	F	IBS	Bisacodyl/PEG 3350 15 mg/2 L	Screening Colonoscopy No NA	6 days	Abdopain and hematochezia	Flexible sigmoidoscopy	None	Conservative	D/C < 1 day (no admission)

Abdopain, abdominal pain; SLE, systemic lupus erythematosus, HTN, hypertension; MCTD, mixed connect tissue disease; NA, not available; D/C, day of discharge; CT, computerized tomography; GAS, group A *Streptococcus*.

## Data Availability

The data generated or analysed during this study are included within this article.
